# Disparate Effects of p24α and p24δ on Secretory Protein Transport and Processing

**DOI:** 10.1371/journal.pone.0000704

**Published:** 2007-08-08

**Authors:** Jeroen R. P. M. Strating, Gerrit Bouw, Theo G. M. Hafmans, Gerard J. M. Martens

**Affiliations:** Department of Molecular Animal Physiology, Nijmegen Centre for Molecular Life Sciences, Institute for Neuroscience, Faculty of Science, Radboud University, Nijmegen, The Netherlands; Duke University Medical Center, United States of America

## Abstract

**Background:**

The p24 family is thought to be somehow involved in endoplasmic reticulum (ER)-to-Golgi protein transport. A subset of the p24 proteins (p24α_3_, -β_1_, -γ_3_ and -δ_2_) is upregulated when *Xenopus laevis* intermediate pituitary melanotrope cells are physiologically activated to produce vast amounts of their major secretory cargo, the prohormone proopiomelanocortin (POMC).

**Methodology/Principal Findings:**

Here we find that transgene expression of p24α_3 _or p24δ_2_ specifically in the *Xenopus* melanotrope cells in both cases causes an effective displacement of the endogenous p24 proteins, resulting in severely distorted p24 systems and disparate melanotrope cell phenotypes. Transgene expression of p24α_3_ greatly reduces POMC transport and leads to accumulation of the prohormone in large, ER-localized electron-dense structures, whereas p24δ_2_-transgenesis does not influence the overall ultrastructure of the cells nor POMC transport and cleavage, but affects the Golgi-based processes of POMC glycomaturation and sulfation.

**Conclusions/Significance:**

Transgenic expression of two distinct p24 family members has disparate effects on secretory pathway functioning, illustrating the specificity and non-redundancy of our transgenic approach. We conclude that members of the p24 family furnish subcompartments of the secretory pathway with specific sets of machinery cargo to provide the proper microenvironments for efficient and correct secretory protein transport and processing.

## Introduction

The secretory pathway consists of a number of distinct membrane-bounded subcompartments that have specialized functions in the process of protein biosynthesis [1]. Proteins that pass through the subcompartments undergo various posttranslational modifications (e.g. glycosylation, sulfation and proteolytic cleavage) that are essential for their biological activity. The biosynthetic process includes the selective packaging of proteins from the endoplasmic reticulum (ER) into vesicles for delivery to the subcompartments. Members of the p24 family of type-I transmembrane proteins are thought to be somehow involved in the trafficking events between the ER and the Golgi [2].

The p24 proteins have been found to be abundantly present in transport vesicles coated with the COPI- or COPII-coat protein complex as well as in ER, intermediate compartment and *cis*-Golgi membranes, whereby they shuttle constantly between these subcompartments (reviewed in [2], [3]). The ∼24K p24 proteins constitute a family that can be subdivided into four subfamilies (p24α, β, γ and δ) [4], with each family member displaying multiple domains conserved from yeast to mammals (reviewed in [2]). In view of their structural resemblance, the various members of the p24 family likely have similar functions. The p24 proteins have been primarily proposed to act as receptors for specific sets of secretory cargo molecules (cargo receptor model) [5]. In this report, “secretory cargo” refers to the biologically active transmembrane and soluble proteins that are transported to the plasma membrane or extracellular matrix as well as to the bioactive soluble proteins that are secreted into the extracellular space, whereas the machinery proteins or lipids that are supplied to subcompartments of the secretory pathway to provide the proper microenvironments for efficient and correct transport and processing of the secretory cargo are designated “machinery cargo”. Studies in yeast lacking one or more p24 proteins have indeed indicated that p24 plays a role in the anterograde transport of some (Gas1p and invertase), but not all (α-factor, acid phosphatase, carboxypeptidase Y, alkaline phosphatase and Gap1p) secretory cargo [5]–[9]. Furthermore, when injected into mammalian cells in culture antibodies against the cytoplasmic tail of p23 (p24δ_1_) inhibited the transport of the secretory cargo protein VSV-G [10]. Based on a variety of functional studies, alternative roles for p24 have been proposed, including its involvement in the biogenesis and proper functioning of transport vesicles and in the organization of membranes of the secretory pathway [10]–[14]. Surprisingly, an octuple yeast knock-out strain lacking all p24 proteins was viable [15], whereas homozygous p23 (p24δ_1_) knock-out mice died early in embryonic development [16]. Thus, studies on the role of p24 in the early secretory pathway have not provided conclusive answers.

In general, the outcome of a functional study is greatly determined by the availability of a well-defined model system. To explore the role of p24 proteins, we therefore decided to use the intermediate pituitary melanotrope cells of the amphibian *Xenopus laevis* as a cell model to study protein transport in a physiological context. The *Xenopus* melanotrope cells produce α-melanophore-stimulating hormone (α-MSH), which mediates the process of background adaptation of the animal. The biosynthetic and secretory activity of the melanotrope cells can be modulated by placing the frogs on a white (inactive melanotrope cells) or black (highly active melanotrope cells) background. The manipulation of the activity of the melanotrope cells is strictly regulated by inhibitory and stimulatory neurons of hypothalamic origin. Upon activation, the melanotrope cells produce and proteolytically cleave vast amounts of the prohormone proopiomelanocortin (POMC), the precursor of a number of bioactive peptides, including α-MSH (reviewed in [17]). In the activated melanotrope cells, proteins upregulated together with POMC are thought to play a role in the biosynthesis of the prohormone [18] and include a subset of p24 proteins, namely p24α_3_, p24β_1_, p24γ_3_ and p24δ_2_. Two other members of the p24 family (p24γ_2_ and p24δ_1_) are expressed in the melanotrope cells as well, but not coordinately with POMC [19]. To examine the role of p24 in the biosynthesis of POMC, we chose to generate and analyze *Xenopus* lines with transgene expression of p24α_3_ or p24δ_2_, i.e. two p24s of the upregulated set. We used a POMC gene promoter fragment to target the expression of the transgenes specifically to the melanotrope cells [20], leaving the regulation of these cells by hypothalamic neurons intact. We find that the p24α_3_- and p24δ_2_-trangenic frogs have distinct melanotrope cell phenotypes in that POMC transport and processing was differently affected, allowing us to conclude that p24α_3_ and p24δ_2_ have non-redundant roles in maintaining the functional and structural integrity of the secretory pathway.

## Results

### Generation of *Xenopus laevis* with stable transgene expression of p24α_3_ or p24δ_2_ specifically in the melanotrope cells

**Figure 1:**
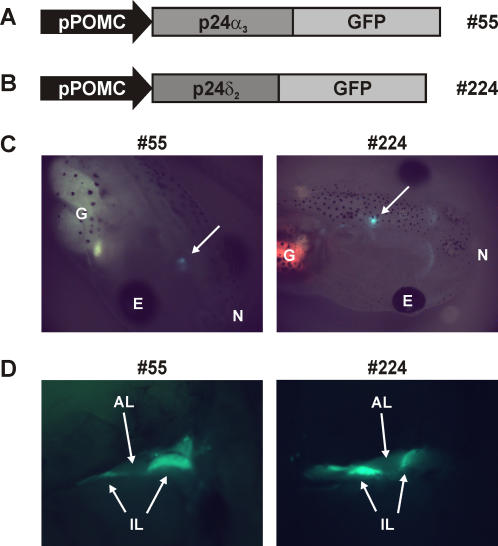
Generation of *Xenopus* with transgene expression of p24α_3_ or p24δ_2_ specifically in the melanotrope cells. (A and B) Schematic representation of the linear injection fragments pPOMC-p24α_3_-GFP (A) and pPOMC-p24δ_2_-GFP (B) containing a *Xenopus* POMC gene promoter fragment (pPOMC) and the protein-coding sequence of p24α_3_-GFP (transgenic lines #605, #55 and #602) or p24δ_2_-GFP (lines #125, #115, #124 and #224); pPOMC drives transgene expression specifically to the melanotrope cells. (C) Pituitary-specific GFP-fluorescence (arrows) in living tadpoles transgenic for p24α_3_ (line #55) or p24δ_2_ (line #224); G, gut; E, eye; N, nose. (D) Fluorescence in the intermediate lobe (IL) and not in the anterior lobe (AL) of the pituitary of adult frogs transgenic for p24α_3_ (#55) or p24δ_2_ (#224).

For our functional studies on p24, we generated *Xenopus laevis* transgenic for the p24α_3_ or the p24δ_2_ protein. In order to drive transgene expression specifically to the melanotrope cells of the *Xenopus* intermediate pituitary, we made DNA-constructs containing a 529-bp *Xenopus laevis* POMC gene A promoter fragment (pPOMC [20]) in front of *Xenopus laevis* p24α_3_ or p24δ_2_ cDNA. To allow direct selection of embryos expressing the transgene, we fused GFP to the C-terminus of p24α_3_ and p24δ_2_. The linearised DNA-constructs (pPOMC-p24α_3_-GFP, Figure 1A or pPOMC-p24δ_2_-GFP, Figure 1B) were mixed with wild-type *Xenopus* sperm nuclei and the mixtures were microinjected into unfertilized wild-type *Xenopus* eggs. We generated three independent transgenic F_0_ frogs for p24α_3_-GFP (#55, #602 and #605) and four independent transgenic F_0_ animals for p24δ_2_-GFP (#115, #124, #125 and #224). Next, F_1_ offspring was produced by *in vitro* fertilization of eggs harvested from wild-type females with sperm isolated from the testes of individual transgenic males or by *in vitro* fertilization of eggs harvested from individual transgenic females with sperm isolated from the testes of wild-type males. Expression of the transgenes specifically in the intermediate pituitary could readily and directly be observed in living tadpoles (Figure 1C) and in adult frogs after lifting the brain (Figure 1D).

**Figure 2:**
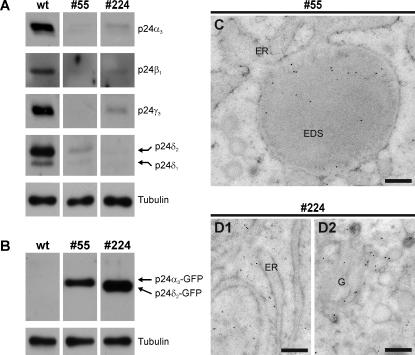
Analysis of p24α_3_ or p24δ_2_ fusion protein expression in transgenic *Xenopus* intermediate pituitary melanotrope cells. (A and B) Western blot analysis of neurointermediate lobe lysates from wild-type frogs (wt) and frogs transgenic for p24α_3_ (#55) or p24δ_2_ (#224) with anti-p24 antibodies (A) or an anti-GFP antibody (B). Tubulin was used as a control for equal loading. (C and D) Immuno-electron microscopy analysis of intermediate pituitary melanotrope cells from frogs transgenic for p24α_3_ (#55; C) or p24δ_2_ (#224; D1 and D2) using an anti-GFP antibody. The p24α_3_-transgene product was mainly present in ER-localized electron-dense structures (EDS; C), whereas the p24δ_2_-transgene product was found on the ER (D1) and on the Golgi (D2). G, Golgi; ER, endoplasmic reticulum. Bars equal 200 nm.

We (in line #124 p24δ_2_-transgenic *Xenopus* [21]) and others (in heterozygous p24 knock-out mice [16], by p24 knock-down in mammalian cells in culture [22], [23] and in p24 knock-out yeast [7], [8]) have observed that manipulation of the expression of a single p24 family member interferes with the endogenous p24 system and affects the steady-state levels of the other p24 family members. We therefore studied in the melanotrope cells of the three p24α_3_- and four p24δ_2_-transgenic *Xenopus* lines the steady-state levels of members of the endogenous p24 protein family. In the melanotrope cells of the three lines transgenic for the p24α_3_-GFP fusion protein, the transgene product effectively displaced the endogenous p24α_3_, β_1_, γ_3_, δ_1_ and δ_2_ proteins (75–90% displacement; n = 4; Figure 2A and data not shown). We selected line #55 to further study the effect of p24α_3_ transgene expression on the functioning of the melanotrope cells. In the transgenic melanotrope cells of line #224, the p24δ_2_-GFP fusion protein displaced the endogenous p24 proteins similarly effective (70–98% displacement; n = 4; Figure 2A). This degree of displacement was higher than that in the previously described p24δ_2_-transgenic melanotrope cells of lines #115, #125 and #124 [21]. We therefore decided to select p24δ_2_-transgenic line #224 for further functional analysis. Surprisingly, whereas the level of expression of the p24α_3_-GFP fusion protein was ∼6 times lower than that of p24δ_2_-GFP (n = 11; Figure 2B), the p24α_3_ and p24δ_2_ fusion proteins were similarly effective in displacing the endogenous p24 proteins.

We next investigated the localization of the fusion proteins within the transgenic melanotrope cells by immuno-electron microscopy (IEM) using an anti-GFP antibody. In the p24α_3_-transgenic melanotrope cells, the majority of the transgene product was found in ER-localized electron-dense structures (EDS, Figure 2C) in which we also observed POMC (see below). In the p24δ_2_-transgenic cells, the gold-label was detected mostly on the ER and the Golgi (Figure 2D), indicating cycling of the fusion protein between these compartments and in line with our previous observation of ER/Golgi localization of the transgene product in line #124 p24δ_2_–transgenic melanotrope cells [21] and consistent with the localization of endogenous p24 proteins (reviewed in [3]).

Together, the studies on the steady-state p24 protein levels showed that in the p24α_3_- and p24δ_2_-transgenic melanotrope cells the entire set of endogenous p24 proteins was nearly completely displaced by the transgene product and therefore the fusion protein was essentially the only p24 protein available to the transgenic cells.

### Steady-state levels of secretory cargo proteins in the transgenic *Xenopus* melanotrope cells

**Figure 3:**
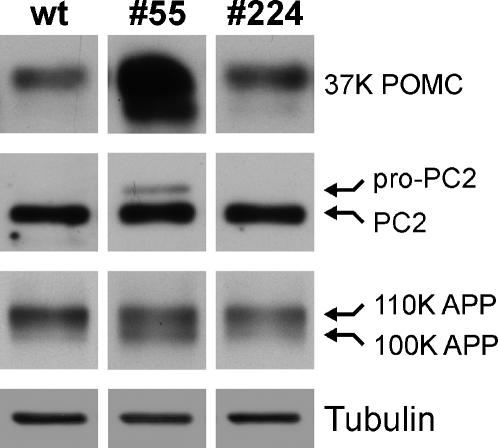
Steady-state levels of secretory cargo proteins in wild-type and transgenic *Xenopus* intermediate pituitary cells. Western blot analysis of neurointermediate lobe (NIL) lysates from wild-type frogs (wt) and frogs transgenic for p24α_3_ (#55) or p24δ_2_ (#224) using antibodies directed against the soluble cargo proteins proopiomelanocortin (POMC) and prohormone convertase 2 (PC2), and the transmembrane cargo amyloid-β precursor protein (APP). Tubulin was used as a control for equal loading.

To study the effect of the transgene expression of p24α_3_ or p24δ_2_ on the functioning of the secretory pathway, we first examined the steady-state levels of the soluble secretory cargo proteins POMC and its processing enzyme prohormone convertase PC2 in the transgenic melanotrope cells. Western blot analysis revealed that the levels of the 37K POMC prohormone were significantly increased in the p24α_3_-transgenic cells (∼11-fold; n = 6), whereas in the p24δ_2_-transgenic cells the steady-state levels of 37K POMC were not affected (n = 6; Figures 3). Furthermore, the proenzyme form of PC2 was detected only in the p24α_3_-transgenic melanotrope cells, and not in wild-type and the p24δ_2_-transgenic cells (Figure 3). We then studied the steady-state levels of a transmembrane cargo protein, namely of the type-I transmembrane protein amyloid-β precursor protein (APP). *Xenopus* APP is synthesized as an ∼100K N-linked glycosylated protein that in the Golgi is converted into an ∼110K O-glycosylated mature protein [24]. Western blot analysis using anti-APP antibodies showed in wild-type cells an ∼5.4-fold (n = 4) higher amount of 110K APP than the level of immature 100K APP. In the p24α_3_-transgenic cells, the level of immature 100K APP was clearly increased (∼1.9-fold; n = 4), such that the ratio between 110K and 100K APP was ∼2.8 (n = 4), whereas the ratio between both forms as well as the total amount of APP were not significantly affected in the p24δ_2_-transgenic cells (n = 4; Figure 3). Thus, increased steady-state levels of the unprocessed forms of both soluble (POMC, pro-PC2) and transmembrane (APP) cargo proteins were detected in the p24α_3_-, but not in the p24δ_2_-, transgenic melanotrope cells. Unfortunately, the use of a battery of antibodies against multiple mammalian ER and Golgi enzymes and matrix proteins did not allow us to determine the steady-state levels of these proteins in the transgenic cells because of the inability of the antibodies to cross-react with the *Xenopus* orthologs.

We next investigated the peptides produced by POMC cleavage through matrix-assisted laser-desorption-ionization time-of-flight mass spectrometry (MALDI-TOF MS). The POMC-derived peptides des-N-α-acetyl-α-MSH (the nonacetylated form of α-MSH), α-MSH, γ_1_-MSH, β-MSH and two corticotrophin-like intermediate lobe peptides (CLIP A and B) were found in wild-type melanotrope cell extracts as well as in samples from the p24α_3_- and p24δ_2_-transgenic cells (data not shown), indicating that both the p24α_3_- and the p24δ_2_-transgenic melanotrope cells converted 37K POMC into the same set of bioactive peptides as the wild-type cells.

### Biosynthesis of POMC in the transgenic *Xenopus* melanotrope cells

**Figure 4:**
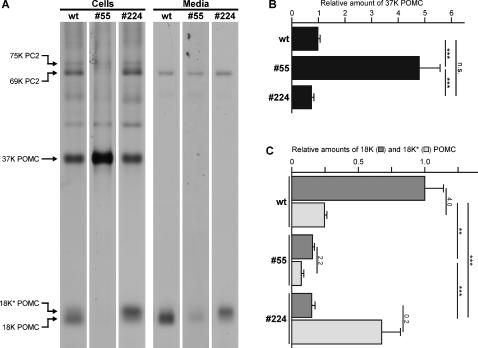
The effect of p24α_3_- or p24δ_2_-transgene expression on POMC biosynthesis and processing in *Xenopus* melanotropes. (A–C) Neurointermediate lobes (NILs) from wild-type frogs (wt) and frogs transgenic for p24α_3_ (#55) or p24δ_2_ (#224) were pulse labeled with [^35^S]-Met/Cys for 30 min and subsequently chased for 3 hrs. Newly synthesized proteins extracted from the NILs (Cells; 5% of extract) and secreted into the incubation medium (Media; 20%) were resolved by 15% SDS-PAGE and visualized by autoradiography. (A) The analysis was performed in six independent experiments and a representative autoradiogram is shown. (B) The amount of newly synthesized 37K POMC in wild-type (n = 16) and the p24α_3_-transgenic (n = 10) and p24δ_2_-transgenic (n = 6) cells was quantified and is shown relative to the wild-type cells. (C) The amounts of newly synthesized 18K and 18K* POMC in wild-type (n = 12) and the p24α_3_-transgenic (n = 5) and p24δ_2_-transgenic (n = 6) cells were quantified and are shown relative to wild-type 18K POMC. Indicated are the 18K/18K* ratios and their statistical evaluations. Data are shown as means +/− SEM. n.s., not significant; **, p<0.01; ***, p<0.001.

In view of the observed differences in the steady-state protein levels, we next wondered whether the biosynthetic activity for POMC production was affected in the transgenic melanotrope cells. From the pituitary (consisting of the pars nervosa, and the anterior and intermediate lobes), the anterior part can be dissected but the pars nervosa is intimately associated with the intermediate pituitary (the neuroendocrine melanotrope cells). For our studies, we therefore used the neurointermediate lobe (NIL; pars nervosa plus intermediate lobe) of the pituitary to study the biosynthetic events in the melanotrope cells. Since the *Xenopus* pars nervosa consists of biosynthetically inactive nerve terminals of hypothalamic origin, the radiolabelled proteins are synthesized exclusively by the melanotrope cells of the intermediate pituitary. Metabolic cell-labeling experiments revealed that following a 30-min pulse the patterns of newly synthesized proteins produced in the p24α_3_- and in the p24δ_2_-transgenic melanotrope cells were similar to that in wild-type cells (data not shown). Thus, protein biosynthesis was not affected in the transgenic melanotrope cells. Following a 30-min pulse and a 3-hr chase period, we observed a clearly higher amount of newly synthesized 37K POMC remaining in the p24α_3_-transgenic than in wild-type cells (∼5-fold; n = 10; Figures 4A and 4B). In contrast, following the pulse-chase incubation the amount of newly synthesized 37K POMC found in the p24δ_2_-transgenic melanotropes was similar to that in wild-type cells (n = 6; Figures 4A and 4B). These observations are in line with the Western blot results showing higher steady-state levels of 37K POMC in the p24α_3_-transgenic melanotrope cells than in wild-type and the p24δ_2_-transgenic cells (Figure 3). Thus, the levels of POMC biosynthesis were not changed in the melanotrope cells of the two transgenic lines, but the extent of POMC processing appeared to be affected in the p24α_3_-transgenic cells.

### Processing of newly synthesized POMC in the transgenic *Xenopus* melanotrope cells

**Figure 5:**
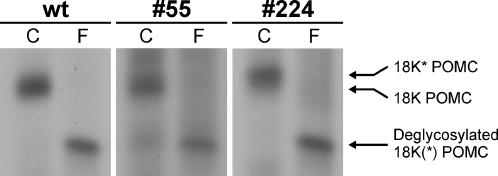
Newly synthesized 18K and 18K* POMC differ in N-glycosylation. Neurointermediate lobes (NILs) from wild-type frogs (wt) and frogs transgenic for p24α_3_ (#55) or p24δ_2_ (#224) were pulse labeled with [^35^S]-Met/Cys for 30 min and subsequently chased for 3 hrs. Newly synthesized proteins extracted from the NILs were deglycosylated with PNGaseF (F) or control-treated (C), resolved by 20% SDS-PAGE and visualized by autoradiography; the #55 lanes were exposed three times longer than the other lanes.

Because of the observed accumulation of POMC in the p24α_3_-transgenic melanotrope cells, we next studied the dynamics of POMC processing by performing pulse-chase metabolic cell labeling experiments. The first endoproteolytic cleavage of newly synthesized 37K POMC yields an ∼18K POMC cleavage product that represents the N-terminal portion of 37K POMC and contains the only N-linked glycosylation site present in the *Xenopus* POMC molecule [25]. In wild-type melanotrope cells, after a 30-min pulse and 3-hr chase period, two forms of 18K POMC are produced, a major product (∼80%) indicated as 18K POMC and a minor (∼20%), slightly slower migrating product that we previously designated 18K* POMC (Figure 4A; [21]). In the p24α_3_-transgenic cells, the amounts of the 18K and the 18K* POMC processing products were clearly reduced (n = 6; Figures 4A and 4C), indicating less proteolytic processing of newly synthesized 37K POMC. In the p24δ_2_-transgenic cells, the level of 18K POMC was greatly diminished (∼7-fold; n = 6), whereas the amount of the 18K* POMC product was clearly increased (∼3-fold; n = 6; Figures 4A and 4C), reducing the ratio 18K/18K* POMC from ∼4 in wild-type cells to ∼0.2 in the p24δ_2_-transgenic cells (Figure 4C). In line #124 p24δ_2_-transgenic cells, we have previously also observed a decrease in the 18K/18K* POMC ratio, albeit less pronounced [21]. The two forms of 18K POMC presumably differ in the sugar moieties added to their N-glycosylation sites, because deglycosylation of the newly synthesized melanotrope cell proteins with peptidyl N-glycosidase F (PNGaseF), an enzyme that removes all N-linked sugar groups from the protein backbone, caused a shift of both 18K and 18K* POMC to an ∼15.5K product (Figure 5). These results suggest that only in the p24δ_2_-, and not in the p24α_3_-, transgenic cells the glycosylation machinery was affected. Next, HPLC analysis was used to study the newly synthesized peptides resulting from the proteolytic cleavage of radiolabelled POMC. This analysis revealed that the proteolytic cleavage of newly synthesized POMC to des-α-MSH, α-MSH, the two forms of CLIP and the two forms of endorphin was similar in the transgenic and wild-type cells (data not shown), in line with the results obtained by mass spectrometry analysis of the steady-state peptides. Together, the biosynthetic studies on the processing of newly synthesized POMC showed that the processing rate was severely reduced in the p24α_3_-transgenic cells and that the glycosylation event was affected in the p24δ_2_-transgenic cells.

### Sulfation of newly synthesized POMC in the transgenic *Xenopus* melanotrope cells

**Figure 6:**
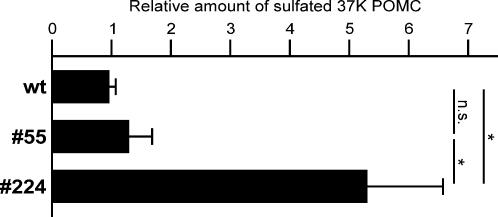
Sulfation of newly synthesized POMC in wild-type and transgenic *Xenopus* intermediate pituitary cells. Neurointermediate lobes (NILs) from wild-type frogs (wt) and frogs transgenic for p24α_3_ (#55) or p24δ_2_ (#224) were pulse labeled with ^35^S-sulfate and ^3^H-lysine for 15 min. Newly synthesized proteins extracted from the NILs were resolved by 15% SDS-PAGE and the amount of [^35^S]SO_4_ and ^3^H-lysine incorporated into newly-synthesized 37K POMC was determined. Shown are the amounts of newly synthesized sulfated 37K POMC produced in the transgenic relative to wt NILs. Data are shown as means +/− SEM (wt, n = 7; transgenics, n = 5). *, p<0.05; n.s., not significant.

In addition to N-linked glycosylation and proteolytic cleavage, POMC is posttranslationally modified by sulfation in the *trans*-Golgi network (TGN) of the *Xenopus* melanotrope cells [26]. To examine whether sulfation of newly synthesized POMC was affected in the transgenic melanotrope cells, we pulse labeled the cells in the presence of both ^3^H-lysine and ^35^S-sulfate. The labeling with ^3^H-lysine allowed us to quantify the total amount of newly synthesized POMC and ^35^S-sulfate labeling revealed the degree of sulfation of the newly synthesized prohormone. 37K POMC sulfation was not affected in the p24α_3_-transgenic cells, whereas we observed an ∼5-fold increase in the sulfation of the prohormone in the p24δ_2_-transgenic cells (Figure 6). Removal of the N-linked glycans from the sulfate-labeled 37K POMC with PNGaseF increased its electrophoretic mobility but did not affect the degree of sulfate labeling (data not shown), suggesting that the sulfate did not reside on the glycogroup. Together with the fact that the 18K POMC species were not sulfated and in line with the presence of a predicted tyrosine sulfation site in the C-terminal half of the *Xenopus laevis* 37K POMC molecule (Sulfinator prediction program [27]), the sulfate label presumably resided on tyrosine residue 188 (SLELD**Y^188^**PEIDLDEDIED). The biosynthetic studies with ^35^S-sulfate thus showed that the level of POMC sulfation in the TGN was increased in the p24δ_2_-, but not the p24α_3_-, transgenic melanotrope cells.

### Electron and immuno-electron microscopy analysis of the transgenic *Xenopus* melanotrope cells

**Figure 7:**
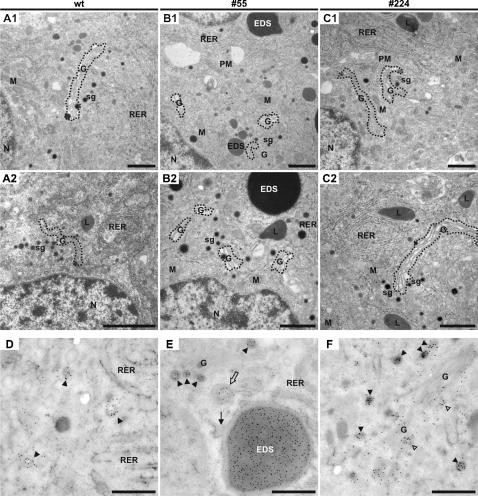
Electron microscopy analysis of wild-type and transgenic melanotrope cells. (A–C) Wild-type (wt; A1 and A2) intermediate pituitary cells showed a well-developed rough endoplasmic reticulum and extensive Golgi-ribbons. The p24α_3_-transgenic cells (#55; B1 and B2) contained Golgi mini-stacks and large electron-dense structures (EDS). The p24δ_2_-transgenic cells (#224; C1 and C2) showed an ultrastructure similar to that of wild-type cells. The dotted lines highlight the outline of the Golgi. (D–F) Immuno-electron microscopy analysis of intermediate pituitary melanotrope cells from wt frogs (D), and frogs transgenic for p24α_3_ (#55; E) or p24δ_2_ (#224; F) using an anti-POMC antiserum. Immunoreactivity was found in dense-core secretory granules (filled arrowheads) in wt and p24α_3_- and p24δ_2_-transgenic cells and occasionally in newly forming secretion granules still attached to the *trans*-Golgi network (open arrowheads). In addition, in the p24α_3_-transgenic cells a strong POMC-immunolabeling was observed in the EDS, which were localized to the ER lumen (arrow) and occasionally in EDS newly forming within the ER lumen (open arrow). G, Golgi; L, lysosome; M, mitochondrion; N, nucleus; RER, rough endoplasmic reticulum; PM, plasma membrane; sg, immature secretory granules. Bars equal 1 µm (A–C); 500 nm (D–F).

In view of the observed differences in POMC transport and processing, we decided to examine the ultrastructure of the transgenic cells by performing EM. Like in wild-type cells, in the p24α_3_- and p24δ_2_-transgenic melanotrope cells the rough ER was well developed. However, the p24α_3_-transgenic cells contained besides the large spherical EDS a Golgi apparatus that appeared as fragmented mini-stacks (Figures 7A and 7B). Despite the fact that the p24 system was also severely distorted, the p24δ_2_-transgenic cells had normal Golgi ribbons and overall no gross morphological differences were observed between these cells and wild-type melanotropes (Figures 7A and 7C). IEM using the anti-POMC antibody revealed in the p24α_3_-transgenic cells strong immunolabeling for POMC in the EDS that were localized within the ER lumen (Figure 7E), indicating that the unprocessed and accumulated prohormone most likely resided in these structures. Furthermore, POMC-labeling was found in dense-core secretory granules of wild-type and the p24α_3_- and p24δ_2_-transgenic melanotrope cells (Figures 7D–F). Together, these results showed that the expression of the p24α_3_-transgene product caused an accumulation of POMC in large electron-dense structures within the ER and impaired the structural integrity of the Golgi apparatus, whereas the transgenic expression of p24δ_2_ did not affect the ultrastructure of the cells.

## Discussion

The type-I transmembrane p24 proteins are abundantly present in ER- and Golgi-derived transport vesicles, and are therefore thought to play an important role in some aspect of cargo-selective transport through the early secretory pathway (reviewed in [2]). The complex and dynamic behavior of this protein family has hampered functional analyses. In this study, we used the *Xenopus laevis* intermediate pituitary melanotrope cells to explore the function of p24. The *Xenopus* melanotrope cells are well-defined neuroendocrine cells of which the biosynthetic and secretory activity can be physiologically induced by placing the frogs on a black background (for review, see [17]). Upon black-background adaptation, four p24 family members (p24α_3_, p24β_1_, p24γ_3_ and p24δ_2_), but not p24γ_2_ and p24δ_1_, are upregulated in the melanotrope cells together with the main secretory cargo protein POMC [19]. The specific upregulation of these four p24 proteins suggests that they are somehow involved in the biosynthesis of POMC. Of the four upregulated p24 proteins, we chose p24α_3_ and p24δ_2_ for our functional analyses and generated three independent stable transgenic lines expressing p24α_3 _and four independent stable p24δ_2_-transgenic lines, using a POMC gene promoter fragment [20] to drive transgene expression specifically to the melanotrope cells.

We showed in the transgenic *Xenopus* melanotrope cells a nearly complete displacement of the endogenous p24α_3_, p24β_1_, p24γ_3_, p24δ_1_ and p24δ_2_ proteins by both the p24α_3_- and the p24δ_2_-transgene product. This displacement effect is not in line with the result of the transient overexpression of TMP21 (p24δ_1_) or p24a (p24β_1_) in cultured tumor cells, which did not influence the steady-state levels of p24a or TMP21 respectively [22]. This apparent discrepancy may have been caused by the transient nature of the overexpression in the tumor cells (in our transgenic system the overexpression was stable). To our knowledge, the effect of stable overexpression of an exogenous p24 protein on the endogenous p24 system has not been studied before. Yet, knockout or knockdown of a p24 member in yeast or mammalian cells has led to a reduction in the levels of other p24 proteins as well [7], [8], [16]. At present, we can only speculate why the endogenous p24 proteins were displaced in the p24α_3_- and p24δ_2_-transgenic *Xenopus* melanotrope cells. For instance, the amount of early secretory pathway membranes that can harbor p24 proteins may be limited and the relative levels of the various newly synthesized p24 family members expressed in a cell will then determine the final composition of the p24 system. In the transgenic melanotrope cells, the relatively high amount of newly synthesized transgene product will occupy most of the available space, consequently leading to the displacement of the endogenous p24 proteins. Alternatively, since p24 protein stability depends on the presence of other p24 proteins and their interaction within p24 complexes [7], [8], [16], and the stable transgene expression in the *Xenopus* melanotrope cells disturbed the stoichiometry of the p24 system, the transgenic manipulation may well have resulted in altered p24 complex compositions. As a consequence, the endogenous p24 proteins would not be able to form their favored and thus stable complexes, leading to the observed reduced endogenous p24 protein levels. Remarkably, for unknown reasons the p24α_3_-transgene product displaced the endogenous p24 proteins more effectively than the p24δ_2_-transgene product.

Irrespective of the explanation, the nearly complete displacement of the endogenous p24 proteins implies that in the p24α_3_- and p24δ_2_-transgenic *Xenopus* melanotrope cells the p24 system consists essentially of only the p24α_3_- and p24δ_2_-transgene product respectively. This unique situation allowed us to search for the role of p24 by analyzing the functioning of the secretory pathway in the transgenic cells. For our functional analysis, we took advantage of the fact that the *Xenopus* melanotrope cells produce and process only one major secretory cargo protein (POMC) and that in wild-type melanotrope cells the biosynthetic processes have been previously analyzed in detail (reviewed in [17]). In the p24α_3_-transgenic melanotrope cells, the amounts of newly synthesized POMC, the level of POMC sulfation as well as the ratio of 18K- to 18K*-POMC were normal, but the rates of transport and proteolytic cleavage of 37K POMC were greatly reduced. The uncleaved POMC accumulated in large electron-dense structures localized to the ER. Since these structures were present only in the p24α_3_- and not in the p24δ_2_-transgenic melanotropes nor in *Xenopus* melanotropes transgenic for other (transmembrane) proteins (our unpublished observations), they were not merely a result of transgenic protein expression *per se*. Furthermore, in the p24α_3_-transgenic cells the Golgi appeared as ministacks, whereas in wild-type and the p24δ_2_-transgenic cells this subcompartment was present as Golgi ribbons. An attractive explanation for these observations is that the excess of p24α_3_ had affected the supply of the machinery cargo that is required for a correct microenvironment in the early secretory pathway with the consequence of an improper transport and accumulation of POMC, and an abnormal structural organization of the Golgi apparatus in the p24α_3_-transgenic cells. In the p24δ_2_-transgenic melanotrope cells, the overall ultrastructure was not affected by the transgenic manipulation and the levels and rates of POMC synthesis, transport and cleavage were normal. Also, the steady-state and biosynthetic levels of the POMC-cleavage enzyme PC2 and the transmembrane cargo APP were similar in wild-type and the p24δ_2_-transgenic cells. However, higher amounts of the differentially glycosylated 18K* POMC-product and of sulfated POMC were found in the p24δ_2_-transgenic than wild-type or the p24α_3_-transgenic cells. Since the processes of glycomaturation and sulfation occur in the Golgi, it appears that excess p24δ_2_ had resulted in an increased supply of the machinery cargo that is responsible for these processes in this subcompartment of the secretory pathway. Taken together, our results suggest that in the p24α_3_- and p24δ_2_-transgenic melanotrope cells the microenvironments of, albeit different, subcompartments in the early stages of the secretory pathway were affected, pointing to a role for p24 proteins in the transport of subsets of machinery cargo and thus in furnishing specific subcompartments of the early secretory pathway.

In view of their structural similarity, the various p24 family members presumably have similar functions. Thus far, the p24 proteins have been mainly implicated in the selection and packaging of secretory cargo proteins (cargo receptor model; reviewed in [2]). However, if the p24 proteins were receptors for secretory cargo, it is not clear why in the *Xenopus* melanotrope cells four p24 proteins (p24α_3_, p24β_1_, p24γ_3_, p24δ_2_) are coordinately expressed with POMC, the major secretory cargo (the prohormone represents >80% of all newly synthesized proteins; [19]). Furthermore, if the four upregulated p24 proteins would function together as a heterotetrameric POMC receptor, one would not have expected to observe such different phenotypes for the p24α_3_- and p24δ_2_-transgenic melanotrope cells. Reasoning along the same lines, the role suggested for p24 in vesicle biogenesis [5] appears to be improbable. Thus, the results of our study do not support a role for p24 in secretory cargo receptor or vesicle functioning, but rather are most consistent with a function in furnishing secretory pathway subcompartments. Our furnishing model is compatible with the results of other studies in which the expression of p24 was also manipulated. First, overexpressing p23 (p24δ_1_) or a p25 (p24α_2_) mutant protein in cultured mammalian tumor cells resulted in the formation of Golgi mini-stacks and highly specialized lipid subdomains, respectively, leading the investigators to conclude that p24 proteins are involved in the formation of membrane subdomains in the early secretory pathway [13], [14]. Second, antibodies against the cytoplasmic tails of p24α_2_, p23 (p24δ_1_) or p24 (p24β_1_) inhibited the formation of transport carriers and cargo transport [10], [12], [28], consistent with a role for p24 in the supply of machinery cargo to ER-subdomains that are capable of forming transport carriers. Third, reducing the expression of Tmp21/p23 (p24δ_1_) in mammalian cells in culture with siRNA led to an effect on the fate of the secretory cargo [22], [23]. Fourth, the finding that in single and multiple yeast p24 knock-outs the vesicular incorporation and maturation of a specific set of secretory cargo proteins was reduced [6]–[9], [15], [29] is reminiscent of what we observed in the p24α_3_-transgenic cells and may thus also be explained by an improper supply of subsets of machinery cargo involved in transport carrier formation or maturation of secretory cargo. In addition, these yeast knock-outs had defects in the retention of the ER-chaperones Kar2p (BiP) and protein disulfide isomerase, which may have been caused by an improper maintenance of the ER-retrieval machinery [8], [15], [29], [30]. Fifth, the injection of antibodies against the cytoplasmic-tail of p23 (p24δ_1_) into cultured mammalian tumor cells inhibited retrograde cargo transport due to a malfunctioning of the ER-retrieval machinery [31]. Indeed, p23 (p24δ_1_) directly interacted with the KDEL-receptor, a component of the retrieval system [32]. Sixth, manipulation of p23 (p24δ_1_) or p25 (p24α_2_) expression levels in cultured mammalian tumor cells affected the ER localization of the phosphatase machinery protein TC48 [33]. Seventh, in *C. elegans* mutated p24 proteins damaged the supply of machinery cargo involved in the quality control system, allowing the transport of a mutant cargo protein out of the ER [34]. Thus, the phenotypes displayed by the various cell types in which p24 expression had been manipulated are consistent with a role for p24 in the supply of machinery cargo to secretory pathway subcompartments. Such a role is supported by the results of our search of the BIOGRID database [35] for physical interactors with yeast p24 proteins revealing that, besides constituents of the COPI- and COPII-coat machinery, a substantial number of the interactors are components of the secretory pathway machinery, like membrane-lipid-synthesizing proteins and N- and O-glycosylation enzymes.

An intriguing extension of our model on the role of p24 concerns the recently proposed mechanism for the anterograde-directed bulk flow transport of secretory cargo with progressive en bloc protrusion from the ER, leading to the formation of large tubulo-saccular structures that transport the cargo to the Golgi apparatus [36]. In the so-called ER-exit sites (ERES) adjacent to the large cargo carriers, the COPII-system would recruit machinery cargo needed to create a microenvironment in the ER and Golgi (e.g. a locally different pH or Ca^2+^-concentration) that facilitates efficient secretory cargo delivery to the newly forming transport carriers, and allows later trafficking and processing events [36]. For the *Xenopus* melanotrope cells, an attractive model for ER-to-Golgi secretory cargo transport may therefore involve an en bloc bulk-flow protrusion of POMC with each of the upregulated p24 family members recruiting a specific set of machinery cargo to the ERES in order to allow efficient transport and subsequent correct processing of POMC in the regulated secretory pathway of these neuroendocrine cells. Our model differs from the current models proposing a role for p24 as receptor for secretory cargo or in vesicle biogenesis, and will serve as a challenging foundation for future p24-research.

In summary, we explored the role of two p24 family members by applying a stable, cell-specific transgenic approach in a well-defined and physiologically relevant neuroendocrine cell model. Since both the p24α_3_- as well as the p24δ_2_-transgenic melanotrope cells showed a nearly complete knockdown of the endogenous p24 proteins and still displayed disparate phenotypes, the observed effects are specific and caused by the transgene products rather than by the absence of the endogenous p24 proteins, indicating the selectivity and non-redundancy of our approach. We conclude that in the p24α_3_-melanotrope cell phenotype an improper furnishing of machinery cargo to the early stages of the secretory pathway hampered transport of secretory cargo from the ER, whereas in the p24δ_2_-phenotype the supply of a different set of machinery cargo influenced post-translational secretory cargo modifications in the Golgi. Collectively, the results are thus most consistent with a role for p24 in furnishing the various subcompartments of the early secretory pathway with specific sets of machinery cargo in order to create the proper microenvironments for efficient and correct secretory cargo transport and processing to eventually generate bioactive proteins that function in the plasma membrane/extracellular matrix or are secreted into the extracellular space as biological signals.

## Materials and Methods

### Animals

South African claw-toed frogs *Xenopus laevis* were bred and reared in the Central Animal Facility of the Radboud University (Nijmegen, The Netherlands). Animals were adapted to a black background for at least three weeks. All animal experiments were carried out in accordance with the European Communities Council Directive 86/609/EEC for animal welfare, permit RBD0166(H10) to generate and house transgenic *Xenopus* and permits RU-DEC 2003-53 and 2007-027 from the animal experiment committee of the Radboud University for the use of *Xenopus laevis* frogs.

### Antibodies

The rabbit polyclonal antibodies against a region in the lumenal part of *Xenopus laevis* p24α_3_, against the C-terminal tail of *Xenopus* p24γ_3_, against part of the lumenal region of *Xenopus* p24δ_1_ (anti-RH6), against portions of the lumenal and C-terminal regions of *Xenopus laevis* p24δ_2_ (anti-1262N and anti-1262C respectively) and against the C-terminal region of *Xenopus* APP (C87) have been described previously [19], [24], [37]. We raised a polyclonal antibody against *Xenopus* p24β_1_ by injecting rabbits with a recombinant protein encompassing the lumenal domain of *Xenopus* p24β_1_ fused to GST. A polyclonal antibody against GFP was obtained from Dr. J. Fransen (NCMLS, Nijmegen, The Netherlands), against *Xenopus* POMC (ST62, recognizing only the precursor form) from Dr. S. Tanaka (Shizuoka University, Japan), against recombinant mature human PC2 from Dr. W.J.M. Van de Ven (University of Leuven, Belgium) and the monoclonal anti-tubulin antibody E7 was obtained from Dr. B. Wieringa (NCMLS, Nijmegen, The Netherlands) [38].

### Generation of Xenopus laevis transgenic for p24α_3_ or p24δ_2_

For the *Xenopus* transgenesis experiments, eggs were harvested from mature wild-type female *Xenopus* injected with 375IU human chorionic gonadotropin hormone (Pregnyl; Organon, Oss, The Netherlands) 18hrs before. The eggs were collected in 1×MMR (0.1M NaCl, 2mM KCl, 1 mM MgCl_2_, 1.5mM CaCl_2_, 5mM HEPES pH7.4) and dejellied in 2% L-cysteine/1×MMR pH8.2. To generate *Xenopus laevis* transgenic for p24α_3_-GFP, a linear 2274-base pair *Sal*I/*Nar*I DNA fragment, containing a 529-base pair *Xenopus* POMC gene A promoter fragment (pPOMC [20]) and a cDNA encoding the *Xenopus* p24α_3_ protein (construct pPOMC-p24α_3_-GFP) with the enhanced green fluorescent protein (GFP) protein fused in frame to its C-terminus (p24α_3_-GFP fusion protein), was used for stable *Xenopus* transgenesis [39], [40]. The linearized transgene DNA fragment was mixed with sperm nuclei prepared as described previously [20], [40], incubated at room temperature (RT) for 15 min and injected into the unfertilized eggs. Normally cleaving embryos were selected at the 4-cell stage and grown in 0.1×MMR/6% Ficoll-400 (Sigma) with 50 µg/ml gentamycin (Gibco/BRL) at 18°C until gastrulation (stage 12 [41]) was reached and then in 0.1×MMR containing gentamycin at RT. In living stage-45 embryos [41] anaesthetized with 0.25 mg/ml MS222 (3-aminobenzoic acid ethyl ester; Sigma) the presence of GFP fluorescence was examined using a Leica MZ FLIII fluorescence stereomicroscope and photographs were taken with a Leica DC200 color camera using the Leica DCviewer software. A number of injection rounds resulted in a number of F_0_ animals expressing the fusion protein at various levels. F_1_ transgenic *Xenopus laevis* offspring were generated by *in vitro* fertilization (IVF), either using pieces of testes from male transgenic frogs and eggs harvested from wild-type females or using pieces of testes from male wild-types and eggs from transgenic females. For IVF, the testes of male *Xenopus* frogs were isolated and incubated for 10 min with a monolayer of eggs harvested from females frogs injected with Pregnyl 18 hr before. 0.1×MMR was added and properly dividing embryos were selected and screened for GFP fluorescence as described above for F_0_ tadpoles. To generate p24δ_2_-transgenic *Xenopus*, a linear 2166-base pair *Sal*I/*Nar*I DNA fragment (construct pPOMC-p24δ_2_-GFP), containing the *Xenopus* POMC gene A promoter (pPOMC [20]) and a cDNA encoding the *Xenopus* p24δ_2_ protein with the enhanced GFP protein fused in frame to its C-terminus (p24δ_2_-GFP fusion protein), was used analogous to the method described above to generate the animals transgenic for p24α_3_-GFP. A number of injection rounds resulted in a number of F_0_ animals transgenic for p24δ_2_-GFP and expressing the fusion protein at various levels. F_1_ transgenic *Xenopus laevis* offspring were generated by IVF, using pieces of testes from male transgenic frogs and eggs harvested from wild-type females.

### Western blot analysis

Western blot analysis was performed as described previously [42]. Proteins on the blot were visualized by enhanced chemiluminescence using the Lumi-lightPLUS Western blotting substrate (Roche), detected in an Epi Chemi II Darkroom equipped with a 12-bit SensiCam CCD camera and quantified using the Labworks 4.0 program (UVP BioImaging systems, Cambridge, United Kingdom).

### MALDI-TOF MS

Freshly dissected neurointermediate lobes (NILs) were homogenized on ice in cold 0.1% tri-fluoroacetic acid (TFA; mass spectrometry grade; Merck, Darmstadt, Germany) and kept on ice for 15 min. Subsequently, cell debris was pelleted by centrifugation (10 min, >15000×*g*, 4°C), and supernatants were collected and diluted in 0.1% TFA. Of the diluted homogenates, 1 µl was mixed with 10 µl of a saturated solution of α-cyano-4-hydroxy-cinnamic acid (CHCA; Sigma) in a 1∶1 (v/v) acetonitrile (Sigma)/0.1% TFA mixture. A volume of 0.5 µl of this mixture was spotted on the sample plate and samples were measured in reflectron mode on a Bruker Biflex III machine (Bruker, FRG). The machine was calibrated with the reference peptides Bradykinin fragment 1–7 (757.3997Da; Sigma), Angiotensin II (1,046.5423Da; Sigma), synthetic peptide P_14_R (1,533.8582Da; Sigma) and human ACTH fragment 18–39 (2,465.1989Da; Sigma).

### Pulse and pulse-chase analysis

For metabolic cell labeling, NILs from wild-type and transgenic *Xenopus* were dissected and preincubated in Ringer's medium (112 mM NaCl, 2mM KCl, 2 mM CaCl_2_, 15 mM HEPES pH7.4, 0.3 mg/ml BSA and 2 mg/ml glucose) at RT for 15 min, pulse labeled in Ringer's medium with 5 mCi/ml Tran^35^S-label (ICN Radiochemicals), chased in Ringer's medium supplemented with 0.5 mM L-methionine for the indicated time periods and homogenized as described previously [43]. For the double-labeling experiments to examine the sulfation of newly synthesized POMC, the NILs were pulse labeled for 15 min with 6.67 mCi/ml Na_2_[^35^S]SO_4_ (ICN Radiochemicals) and 1mCi/ml L-[4,5-^3^H]Lysine monohydrochloride (Amersham Biosciences), rinsed briefly to remove free label and homogenized. Parts of the lysates and chase media were analyzed directly on SDS-PAGE, while the remainder was used for Western blot or HPLC analyses. For quantification, the radioactively double-labeled NIL proteins were separated by SDS-PAGE, and slices (2 mm×1 cm) were cut from the gel, incubated overnight with 1 ml 35% H_2_O_2_ (Acros Organics) at 70°C, 4 ml of OptiPhase ‘HiSafe’ 3 counting liquid (Perkin Elmer Life Sciences) was added, ^3^H- and ^35^S-dpm were counted on a Wallac 1410 Liquid Scintillation Counter (Pharmacia) and the amount of incorporated [^35^S]SO_4_ relative to the amount of incorporated ^3^H-lysine was determined for the newly synthesized POMC.

N-linked glycogroups were removed from the newly synthesized NIL proteins by boiling the homogenates in 6 mM HEPES/0.06% SDS pH7.4 for 10 min, cooling to room temperature, adding 1 µl 12.5% NP40/2.5 mM phenylmethylsulphonyl fluoride (PMSF)/0.25 mg/ml trypsin inhibitor and 1 U Peptidyl N-glycosidase F (PNGaseF; Roche) and incubating the samples overnight at 37°C. The deglycosylated proteins were resolved by 20% SDS-PAGE and visualized by autoradiography.

### HPLC

For the separation of the newly synthesized POMC-derived peptides, radiolabelled NIL lysates were subjected to HPLC analysis as described previously [44].

### Transmission electron microscopy

For ultrastructural studies, NILs were freshly isolated from wild-type and transgenic *Xenopus* and fixed overnight at 4°C in 2% glutaraldehyde in 0.1 M phosphate buffer (PB, pH7.3). After rinsing in the same buffer, fixed tissues were osmicated for one hour in 1% osmium tetroxide in 0.1 M PB, rinsed in PB, dehydrated through graded series of alcohol and embedded in Epon 812. One-micron thick sections were cut, stained with toluidine blue and examined in a phase-contrast microscope (Dialux 20, Leitz). Ultrathin sections were cut, double contrasted with uranyl-acetate/lead-citrate and photographed using a transmission electron microscope (JEOL1010).

### Immuno-electron microscopy

For freeze substitution and low-temperature embedding, the tissue was rapidly frozen by a Leica EM High-Pressure Freezing system (HPF, Leica Microsystems) and transferred to the precooled chamber (−90°C) of a CS auto freeze substitution apparatus (Reichert-Jung, Germany). Freeze substitution was performed according to standard procedures. The tissue was immersed for 72 hours in acetone containing 0.5% uranyl acetate as fixing agent at −90°C and the temperature was then raised stepwise 4°C per hour to −45°C. Prior to infiltration with Lowicryl HM20 resin (Bio-Rad Richmond, California; USA), the tissue was washed several times with acetone at −45°C to remove water and excess uranyl acetate. The embedding process was carried out at −45°C in three stages, with a progressively increasing ratio of resin to acetone. Diffuse UV-radiation (360nm) was used to catalyze polymerization first at −45°C overnight and then at room temperature for 24 hrs. Thin sections were cut on a Reichert Ultracut-E and mounted on one-hole nickel grids coated with a formvar film.

For postembedding immunohistochemistry, ultrathin Lowicryl sections were washed for 10 min in phosphate buffered saline (PBS, pH 7.4) containing 0.1% sodium borohydride and 50 mM glycine, and for 10 min in PBS containing 0.5% BSA and 0.1% cold fish skin gelatine (PBG). For immunolabeling, sections were incubated overnight at 4°C in drops of PBG containing anti-GFP or anti-POMC antibody. Sections were washed for 20 min in PBG, incubated with protein A-labeled 10 nm gold markers, washed in PBS and postfixed with 2.5% glutaraldehyde in PB for 5 min to minimize loss of gold label during the contrasting steps. After washing with distilled water, sections were contrasted in uranyl acetate and studied using a JEOL1010 TEM electron microscope.

### Quantification and statistics

Statistical evaluation was performed using unpaired two-tailed *t*-tests. In those cases where the variances were significantly different, Welch's correction was used. Calculations were performed using the GraphPad Prism 4 program (GraphPad Software).
